# Recent developments in the role of protocatechuic acid in neurodegenerative disorders

**DOI:** 10.17179/excli2023-5940

**Published:** 2023-07-07

**Authors:** Riya Thapa, Ahsas Goyal, Gaurav Gupta, Asif Ahmad Bhat, Santosh Kumar Singh, Vetriselvan Subramaniyan, Sanjay Sharma, Parteek Prasher, Vikas Jakhmola, Sachin Kumar Singh, Kamal Dua

**Affiliations:** 1 School of Pharmacy, Suresh Gyan Vihar University, Jagatpura, Mahal Road, Jaipur, India; 2 Institute of Pharmaceutical Research, GLA University, Mathura, U.P., India; 3 Center for Transdisciplinary Research, Saveetha Institute of Medical and Technical Science, Saveetha University, Chennai, India; 4 Pharmacology Unit, Jeffrey Cheah School of Medicine and Health Sciences, Monash University, Malaysia, Jalan Lagoon Selatan, Bandar Sunway, 47500 Selangor Darul Ehsan, Malaysia; 5 Shobhaben Pratapbhai Patel School of Pharmacy and Technology Management, SVKM'S NMIMS, V. L. Mehta Road, Vile Parle (W), Mumbai, India; 6 Department of Chemistry, University of Petroleum & Energy Studies, Energy Acres, Dehradun 248007, India; 7 Uttaranchal Institute of Pharmaceutical Sciences, Uttaranchal University, Dehradun 248007, India; 8 School of Pharmaceutical Sciences, Lovely Professional University, Phagwara 144411, India; 9 Faculty of Health, Australian Research Centre in Complementary and Integrative Medicine, University of Technology, Sydney, Ultimo-NSW 2007, Australia; 10 Discipline of Pharmacy, Graduate School of Health, University of Technology, Sydney, Ultimo-NSW 2007, Australia

## ⁯

Neurodegenerative disorders are chronic conditions characterized by the progressive and consistent loss of nerve cells in the brain and spinal cord. Diseases like Alzheimer's, Parkinson's, and Huntington's are prominent contributors to the cognitive, motor, and behavioral decline associated with aging. As no cure has yet been discovered, present endeavors primarily concentrate on alleviating symptoms and delaying the advancement of these illnesses. Therefore, it is necessary to develop medications with multiple biological functions that can influence the various alterations in the nervous system, which contribute to the onset and progression of CNS diseases. Consequently, it is important to develop medications with a wide range of biological capabilities that can affect changes in the nervous system, thereby contributing to the development and progression of neurodegenerative disorders (Thapa et al., 2022[18]). Parkinson's disease (PD) and Alzheimer's disease (AD) are among the many neurodegenerative diseases whose incidence is rapidly increasing. Dementia is a major global health concern. According to estimates by the World Health Organization (WHO), around 50 million people worldwide were living with dementia in 2020. This number is projected to increase to 82 million by 2030 and 152 million by 2050. The prevalence of dementia increases with age, and it is more common in older adults. Despite making significant progress, we have not yet developed viable treatments for AD and other neurodegenerative illnesses. Additionally, we face a lack of good early-detection indicators for these diseases (Zheng and Chen, 2022[23]). Evidence of this extent demonstrates the urgent need to address the threat of neurodegenerative disorders to global human health. 

There is an increasing tendency in the pharmaceutical industry to utilize naturally sourced materials for the development of medications that can effectively address neurodegenerative diseases. Within this category of naturally derived substances, protocatechuic acid (PCA) is gaining significant attention and recognition. Chemically, protocatechuic acid (PCA) is known as 3,4-dihydroxybenzoic acid, a phenolic acid that occurs naturally (Kakkar and Bais, 2014[10]). Because of its wide range of anti-inflammatory and antioxidant properties, it possesses a diverse range of potential therapeutic uses. The number of published studies on protocatechuic acid (PCA) has increased, emphasizing the importance of understanding the pharmacological applications of PCA (Kakkar and Bais, 2014[10]). Experimental studies present compelling evidence indicating that PCA may offer protection against neurodegenerative disorders such as AD and PD. This protection is achieved through the inhibition of β-amyloid plaque buildup in the CNS, excessive generation of ROS, hyperphosphorylation of neuronal τ proteins, and neuroinflammation. These factors significantly contribute to cognitive and behavioral decline. Consequently, PCA has the potential to become a safe and effective medication for preventing neurodegenerative diseases in the future (Krzysztoforska et al., 2019[12]; Rawat et al., 2020[15]). AD is marked by cognitive and behavioral decline that significantly restricts an individual's capacity to engage in social and professional activities. The disease has an extended preclinical phase, progresses gradually, and presents initial symptoms such as memory loss, reduced language abilities, mood fluctuations, anxiety, and irritability (Castellani et al., 2010[3]; Rawat et al., 2022[15]). Song et al. assessed the protective effects of PCA in diminishing amyloid deposition and inflammation in aged AD-model mice with cognitive impairments. In PCA-treated AβPP/PS1 animals, there was a significant increase in BDNF levels in both the hippocampus and cerebral cortex. The findings demonstrate that PCA effectively reduced β-amyloid and AβPP levels, as well as the inflammatory response. Additionally, PCA elevated BDNF levels, which are associated with improved cognitive function and memory, while also reducing the inflammatory response (Song et al., 2014[17]). Similarly, Choi et al. conducted a study to assess how effective PCA is in preventing cognitive decline in a mouse model of AD induced by amyloid-β. The findings demonstrated that administering higher amounts of PCA successfully prevented cognitive impairment. Furthermore, the PCA-treated groups exhibited reduced neuroinflammation caused by β-amyloid, as evidenced by lower levels of inflammatory mediators like iNOS and COX-2. Consequently, the researchers concluded that PCA might serve as a protective factor against AD (Choi et al., 2020[4]). Ban et al. found that PCA inhibits neurotoxicity generated by β-amyloid peptide and protects against neuronal cell death. The caspase 3 levels which were increased in brain of AD patients, has been studied to show a crucial role in apoptosis induced by β-amyloid. The results have shown that PCA reduced release of glutamate, the formation of ROS, and caspase 3 activation (Ban et al., 2006[2]). Li et al. examined the primary protective effects and mechanisms of PCA, a significant metabolite of BBE for activating autophagy and, by extension, playing a neuroprotective function. Their results showed that neuronal morphological damage was mitigated in Aβ PP/PS1 animals treated with BBE, and autophagy-related protein expression was increased. PCA successfully reversed the cytotoxicity caused by β-amyloid *in vitro*, including reduced neuron viability and elevated levels of LDH and ROS (Li et al., 2022[13]; Pathak et al., 2021[14]).

PD is a common type of neurodegeneration found in humans. The degenerative effects of PD specifically target dopaminergic neurotransmission in the substantia nigra, making it particularly vulnerable. PD is characterized as a chronic and progressive neurodegenerative condition (Han et al., 2018[9]). Several factors, such as genetics, advanced age, oxidative stress, and mitochondrial dysfunction, have all been linked to the development of PD. Wu et al. conducted an evaluation of the role of PCA in the treatment of PD. They selected PCA to be combined with Ginkgolide B (GB). The findings demonstrated that a treatment strategy combining GB and PCA significantly improved motor function, reduced nerve cell damage, increased antioxidant enzyme activity in brain tissue, and elevated TH expression in the substantia nigra (SN) of midbrain in PD mice. This concept of enhancing the effectiveness of GB in treating PD represents a novel approach for maximizing the utilization of active components within GB (Wu et al., 2020[19]). The loss of dopaminergic neurons in the SN and the presence of α-synuclein inclusions are both indicators of PD. Zhang et al. conducted a study in which they examined the effects of PAC on MPTP-induced reductions in the rotarod latent period and levels of dopamine (DA) and DA metabolites in the striatum. They also investigated how PAC improved MPTP-induced pathology in the SN and reversed the decreases in SN tyrosine hydroxylase expression in C57BL/6J mice. Their findings provide the initial evidence that PAC exhibits neuroprotective properties in mice treated with MPTP, suggesting its potential usefulness in the therapeutic management of PD in humans (Gupta et al., 2018[8]; Zhang et al., 2010[21]). In other studies, Zhang et al. demonstrated that the involvement of α-synuclein aggregation into oligomeric species in the pathogenesis of PD can be inferred from its impact on neuronal survival. Reports have indicated that PAC may protect neurons. Their findings revealed that PAC prevented aberrant oligomerization of α-synuclein, downregulation of TH expression, apoptotic morphology, and cytotoxicity in MPP(+) treated PC12 cells. These results suggest that the neuroprotective effects of PAC on MPP(+)-treated PC12 cells are linked to its ability to prevent α-synuclein oligomerization (Gupta et al., 2021[7]; Zhang et al., 2009[20]). Guan et al. investigated the impact of PCA on apoptotic cell death and mitochondrial dysfunction induced by MPP(+) in PC12 cells. The reduction of Bcl-2, activation of caspase-3, depletion of GSH, production of ROS, and loss of mitochondrial membrane potential were identified as contributing factors to MPP(+)-induced apoptosis in PC12 cells. However, PCA therapy effectively mitigated the aforementioned mitochondrial malfunction in PC12 cells. These findings highlight the potential therapeutic application of PCA for treating neurodegenerative conditions such as PD (Guan et al., 2006[5]). In the same way, oxidative stress and apoptosis caused by MPP(+) were decreased in mature PC12 cells through the use of an Alpinia oxyphylla ethyl acetate extract. An et al. illustrated in their study that PCA exhibits significant protective potential against MPP(+)-induced apoptosis in PC12 cells. Furthermore, it elevated the activity of catalase and superoxide dismutase in PC12 cells. Additionally, PCA reduced cell mortality induced by sodium nitroprusside or H_2_O_2_ in PC12 cells, thus providing a beneficial therapeutic approach for managing neurodegenerative diseases such as PD (An et al., 2006[1]; Gupta et al., 2022[6]). Poly-pharmacology-based techniques, employing combinations of medications with diverse mechanisms of action, offer a distinctive strategy for discovering potential treatments for neurodegenerative diseases. Zhang et al. examined the combined neuroprotective impacts of chrysin and PCA. The outcomes of their study demonstrated that these two polyphenols had synergistic neuroprotective effects, with chrysin enhancing the advantages of PCA. Consequently, PC12 cells treated with 6-hydroxydopamine exhibited increased cell survival rates and reduced LDH release. Moreover, the treatment involving PCA and chrysin effectively lowered the expression of iNOS and NF-kB (Zhang et al., 2015[22]). Two severe symptoms of amyotrophic lateral sclerosis (ALS) are skeletal muscle atrophy and motor neuron apoptosis, which result from neuroinflammation, mitochondrial malfunction, nitrosative stress, and oxidative stress. Koza et al. conducted a study to explore the potential of PCA as a treatment for ALS in transgenic mice expressing a mutant form of the human Cu, Zn-SOD 1. The researchers observed that PCA decreased microgliosis and astrogliosis in the spinal cord of transgenic mice, protected motor neurons from apoptosis, and maintained the integrity of the neuromuscular junction. In this mouse model of ALS, PCA extended lifespan, alleviated clinical symptoms, and slowed down the progression of the disease (Koza et al., 2020[11]). 

Protocatechuic acid has shown promising potential in the field of neurodegenerative disorders. Extensive research has highlighted its ability to exert neuroprotective effects through various mechanisms, including antioxidant, anti-inflammatory, and anti-apoptotic activities. PCA has demonstrated its ability to mitigate oxidative stress, reduce neuroinflammation, and enhance neuronal survival in several *in vitro *and* in vivo* studies. Further research is needed to fully understand its mechanisms of action and optimize its therapeutic potential. Clinical trials are essential to assess the safety and efficacy of PCA in human subjects. Additionally, exploring the synergistic effects of PCA with other neuroprotective agents or conventional therapies may enhance its therapeutic efficacy. Moreover, advancements in drug delivery systems can facilitate targeted delivery of PCA to the CNS, improving its bioavailability and therapeutic outcome. Combination therapies that integrate PCA with lifestyle interventions, such as exercise and diet modifications, may provide a holistic approach in managing neurodegenerative disorders.

## Conflict of interest

The authors declare no conflict of interest.
